# Semiempirical Two-Dimensional Model of the Bipolar Resistive Switching Process in Si-NCs/SiO_2_ Multilayers

**DOI:** 10.3390/nano13142124

**Published:** 2023-07-21

**Authors:** Juan Ramirez-Rios, Karla Esther González-Flores, José Juan Avilés-Bravo, Sergio Alfonso Pérez-García, Javier Flores-Méndez, Mario Moreno-Moreno, Alfredo Morales-Sánchez

**Affiliations:** 1 Electronics Department, Instituto Nacional de Astrofísica, Óptica y Electrónica, San Andrés Cholula 72840, Puebla, Mexico; juan.ramirez@inaoep.mx (J.R.-R.); karla.gonzaflores@gmail.com (K.E.G.-F.); juan18ixi@gmail.com (J.J.A.-B.); mmoreno@inaoep.mx (M.M.-M.); 2 Centro de Investigación en Materiales Avanzados S.C., Unidad Monterrey, Parque de Investigación e Innovación Tecnológica (PIIT), Apodaca 66628, Nuevo León, Mexico; alfonso.perez@cimav.edu.mx; 3 Tecnológico Nacional de México/I.T. Puebla-División de Estudios de Posgrado e Investigación, Av. Tecnológico No. 420, Maravillas 72220, Puebla, Mexico; javier.flores@puebla.tecnm.mx; 4 Área de Ingeniería—Benemérita Universidad Autónoma de Puebla, Ciudad Universitaria, Blvd. Valsequillo y Esquina, Av. San Claudio s/n, Col. San Manuel 72570, Puebla, Mexico

**Keywords:** resistive switching memory, conductive filaments, silicon nanocrystals, two-dimensional oxygen vacancy configuration, valence change memory

## Abstract

In this work, the SET and RESET processes of bipolar resistive switching memories with silicon nanocrystals (Si-NCs) embedded in an oxide matrix is simulated by a stochastic model. This model is based on the estimation of two-dimensional oxygen vacancy configurations and their relationship with the resistive state. The simulation data are compared with the experimental current-voltage data of Si-NCs/Si$O_{2}$ multilayer-based memristor devices. Devices with 1 and 3 Si-NCs/Si$O_{2}$ bilayers were analyzed. The Si-NCs are assumed as agglomerates of fixed oxygen vacancies, which promote the formation of conductive filaments (CFs) through the multilayer according to the simulations. In fact, an intermediate resistive state was observed in the forming process (experimental and simulated) of the 3-BL device, which is explained by the preferential generation of oxygen vacancies in the sites that form the complete CFs, through Si-NCs.

## 1. Introduction

In recent years, the study of resistive switching (RS) memories (RSM) has gained importance because of its application in non-volatile memories. Some features of RSM, compared to devices based on floating gate memories, are low electrical power consumption during writing, small size, fast operation [1] and the crossbar architecture of RSM arrays that enables less fabrication steps [2]. Nevertheless, multiple resistive states, memory window, repeatability and endurance are some other characteristics that need to be improved for RS devices. Therefore, these challenges need to be studied through the modeling of the physicochemical phenomena [3].

The typical structures for RSM are based on metal-oxide-metal (MOM) capacitive structure or metal-oxide-semiconductor (MOS)-like devices. The application of an electrical potential to these RSM produce a soft electrical breakdown that allows the formation of some conductive filaments (CF) in the RS materials, switching (FORMING, SET) the device from a high resistive state (HRS, OFF) to a low resistive state (LRS, ON). Once the ON state is activated, the RSM can return to the OFF state in the same polarity (unipolar RS) or by changing the polarity (bipolar RS) if the CF is broken [4].

Different materials such as Hf$O_{2}$, ZnO, Ti$O_{2}$, ${\mathrm{SiN}}_{x}-\mathrm{Pt}$, Sn$O_{2}$, ${\mathrm{NbO}}_{x}$ or ${\mathrm{Hf}}_{0.5}{\mathrm{Zr}}_{0.5}O_{2}$ have been reported as active layers in RSM devices [5,6,7,8,9,10,11], but the implementation of materials used in the actual semiconductor industry, i.e., Si or Si$O_{2}$, needs to be investigated. In 2010, Yao et al. reported the RS properties of a ${\mathrm{SiO}}_{x}$-based device [12] and it was related to the deoxidation–oxidation process of oxygen vacancies that compound the CFs, which results in a change of the valence state of silicon atoms [13]. RS devices with the formation of a metallic filament by the diffusion of metallic ions from electrodes have been also reported in Cu/${\mathrm{SiO}}_{x}$/W devices [14]. Devices with the deoxidation–oxidation process are known as valence change memory (VCM) while those with diffusion of metallic ions are known as electrochemical metallization memory (ECM) [15]. Some works have demonstrated RS properties in ${\mathrm{SiO}}_{x}$ (x<2) films, where the CF was formed by the connection of Si-nanocrystals (Si-NCs) embedded in the ${\mathrm{SiO}}_{x}$ film, corresponding to VCM [16,17].

In previous works, we reported the experimental RS properties of Si-NCs through the use of Si/SiO${}_{2}$ multilayer (ML) structures where the Si-NCs act as conductive nodes that control the CF formation [18,19]. In a recent study on the bipolar RS behavior for Si-NCs/SiO${}_{2}$ ML, the following was observed: if the FORMING process is obtained at reverse bias (positive voltage to the upper electrode), the RS shows an asymmetric current-voltage (I–V) curve, while a symmetric I-V curve is obtained for a forward bias (negative voltage to the upper electrode), the latter being similar to MOM devices [20]. The RS behavior obtained in this kind of device is related to the composition and structure of the Si/SiO${}_{2}$ ML. X-ray photoelectron spectroscopy (XPS) and high resolution transmission electron microscopy (HRTEM) revealed the formation of Si-NCs surrounded by ${\mathrm{SiO}}_{x}$ (x<2) layers. The analysis of the XPS-Si2p spectra measured in the ${\mathrm{SiO}}_{x}$ films that separate the Si-NCs shows a pronounced presence of Si-suboxides, predominantly in the form of the $O_{3}\equiv \mathrm{Si}-\mathrm{Si}$ species (Si${}^{3+}$), which has been reported as a sub-oxide that favors the field-induced formation of oxygen vacancies ($V_{O}$) due to its asymmetrical nature [18,19]. The presence of Si-NCs and the different ${\mathrm{Si}}^{n+}$ suboxides (*n* = 1–3), which are oxygen vacancies, influence the formation and annihilation of the CF and therefore the SET/RESET processes. Therefore, the simulation of the electrical properties of Si-NCs with these Si-suboxides, considered as oxygen vacancies, within the ${\mathrm{SiO}}_{x}$ films is required to understand the different FORMING, SET and RESET processes observed in the RS behavior.

There are models that can explain the RS phenomena in different active materials. From the computationally complex to simple, the RS models can be classified as the following [4]: (i) Ab initio methods that model the ion transport during the formation of CFs [21] and the calculation of the energy states properties due to $V_{O}$[22]; (ii) finite element methods that can use the properties obtained from Ab initio simulations. These models can simulate the generation and recombination between oxygen ions ($O_{\mathrm{ions}}$) and $V_{O}$ by differential equations and their role for the electrical current [23,24,25]; (iii) Semiempirical models that simplify the computational complexity of the Ab initio methods by physical approximation of the formation of CFs and charge transport through it. These models use physical and chemical properties of the materials that could be obtained from method (i) and from experimental results of RS devices [6,26,27,28]; and (iv) Compact models that can use the memristor theory [29] or conceptual simplifications to simulate and describe the RS in electrical circuits. These models take ideas like Leon Chua’s memristor relations [30], cylindrical CFs [31,32], hourglass quantum-point contact [33] or random circuit breaker network [34]. The (i) and (ii) models can require specialized software and their accuracy depends on the computational power and the relatively large time of calculation. In the case of models (iii) and (iv), the reduction of computational complexity is an important advantage and they have demonstrated a good approximation to experimental data [4]. The compact models (model (iv)) simulate individual CFs or the relation between electrical charge and magnetic flux to implement them in circuit simulation; nevertheless, these approximations do not allow for the consideration of specific features of oxide materials such as nanoclusters, which can act as charge traps.

## 2. Materials and Methods

**Figure 1:**
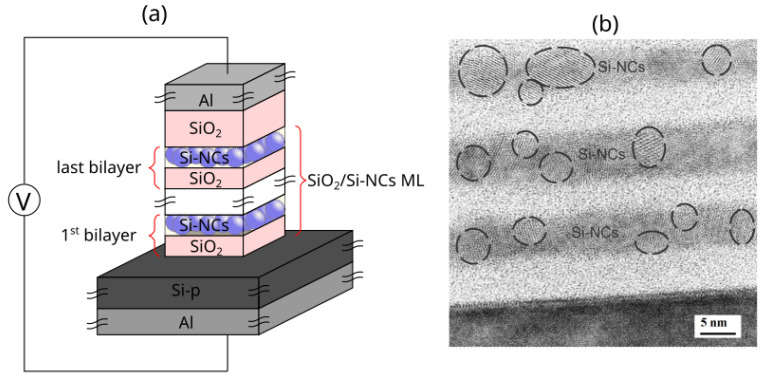
(**a**) Schematic structure (not at scale) and (**b**) HRTEM of an Si-NCs/SiO${}_{2}$ ML-based device.

In this work, we use a semiempirical two-dimensional (2-D) model based on the VCM in relation with the deoxidation–oxidation hypothesis [26] to emulate the bipolar RS properties of Si-NCs/Si$O_{2}$ ML structures where Si-NCs are assumed to be fixed $V_{O}$ that cannot be recombined with $O_{\mathrm{ions}}$. These devices are formed with Si/Si$O_{2}$ bilayers deposited by RF-Sputtering on p-type silicon substrate (1–5 Ω-cm) with Al electrodes (Figure 1a). The presence of the Si-NCs/Si$O_{2}$ ML structure is confirmed by the HRTEM micrograph, shown in Figure 1b. Therefore, the simulation of these devices at different values of voltage sweeps allowed us to obtain, under this assumption, the position of $V_{O}$ generated in a 2-D mesh. These oxygen vacancy configurations ($V_{O}$C) can be used to estimate the resistive state based on the length and number of CFs formed during bipolar RS [35].

## 3. Bipolar Resistive Switching Based on Deoxidation–Oxidation Model

From the rigid ion-point model for ionic crystals [36], and since the Si-O bonds can have an ionic nature [37], the drift velocity *v* of $O_{\mathrm{ions}}$ at high electric fields in oxide thin films can be defined as follows:

(1) $$v=\frac{a}{t_{0}}exp\left(-\frac{E_{Om}}{kT_{J}}\right)sinh\left[\frac{q\varphi_{drift}a\left(-F^{H}\right)}{kT_{J}}\right]$$

where *a* is the mesh size of the 2-D simulation, and it is assumed to be the distance between the neighboring barriers for the drift of $O_{\mathrm{ions}}$, $1/t_{0}$ is the vibration frequency of $O_{\mathrm{ions}}$, $E_{Om}$ is the height of the potential barriers during the migration of $O_{\mathrm{ions}}$, *k* is the Boltzmann constant, $T_{J}$ is the temperature due to the Joule heating, *q* is the fundamental charge, $\varphi_{drift}$ is an enhancement coefficient for the drift of $O_{\mathrm{ions}}$, and $F^{H}$ is the electric field assumed as homogeneous in the active layer. The direction of $F^{H}$ depends on the electrical polarity and it is normal to the surface of the substrate with a magnitude equal to $\left|V_{L}-V_{R}\right|/L$ where *L* is the thickness of the whole active layer. Therefore, the migration of $O_{\mathrm{ions}}$ is opposite to the $F^{H}$. The temperature $T_{J}$ is assumed to be $T_{J}=T_{r}+\left|\left(V_{L}-V_{R}\right)I\right|R_{th}$, with $T_{r}$ as the room temperature, $\left(V_{L}-V_{R}\right)$ the potential difference between the electrodes, *I* the device current and $R_{th}$ the thermal resistance of the CFs [38].

**Figure 2:**
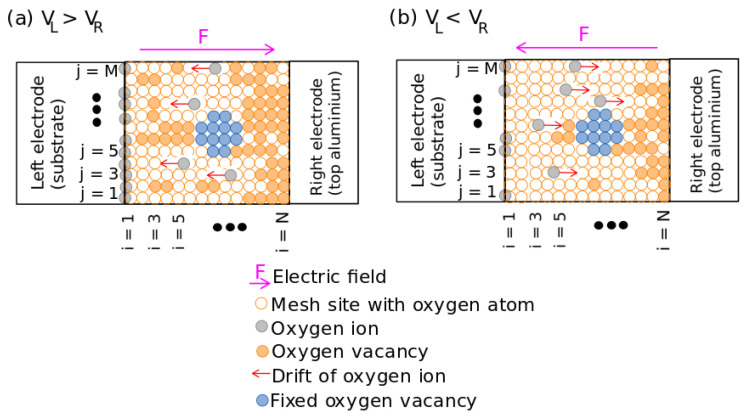
(**a**) Generation of oxygen vacancies and their (**b**) recombination. It is based on the deoxidation–oxidation model where oxygen ions drift into an oxide due to the electric potential difference between left ($V_{L}$) and right electrode ($V_{R}$).

Figure 2 shows the $V_{O}$Cs due to the deoxidation–oxidation process to emulate the bipolar RS process of the Si-NCs/Si$O_{2}$-based RSM. Due to the electric field *F*, the $O_{\mathrm{ions}}$ (gray circle) are pulled up from the equilibrium position in the mesh, drifting at velocity *v* and piling them up at the substrate–oxide interface, as shown in Figure 2a. The sites left by these oxygen atoms generate $V_{O}$ (orange sites), decreasing the valence state of near neighboring silicon atoms as well as reducing the x value of ${\mathrm{SiO}}_{x}$ and then increasing their metallic behavior [13]. These chains of $V_{O}$ can connect the electrodes through Si-NCs (blue sites) and contribute to obtain the LRS (Figure 2a). Since the $O_{\mathrm{ions}}$ are accumulated near the substrate, when an opposite differential potential is applied, these ions return to the ${\mathrm{SiO}}_{x}$ and some $V_{O}$s on the left side of the active layer are recombined allowing that the device returns to the HRS (Figure 2b) [39].

For a time *t*, the generation probability of $V_{O}$ ($P_{ij}^{G}$) and the recombination probability between $O_{\mathrm{ions}}$ and $V_{O}$ ($P_{ij}^{R}$) on each 2-D mesh site (i,j) are [26]:

(2) $$P_{ij}^{G}=\frac{t}{t_{0}}\exp\left[-\frac{E_{Oe}-q\gamma aF_{ij}^{nH}}{kT_{r}}\right]$$

(3) $$P_{ij}^{R}=\frac{t}{t_{0}}\beta_{R}f_{i}\exp\left[-\left(\frac{\left|v\right|\;t}{L_{O}}+\frac{E_{Oe}}{kT_{J}}\right)\right]$$

where $E_{Oe}$ is the migration barrier of the equilibrium position of oxygen ions in the lattice, $F_{ij}^{nH}$ is the non-homogeneous electric field at each mesh site (i,j) (Figure 2), γ is an enhancement coefficient with a different value for the SET and RESET process, $\beta_{R}$ is a constant coefficient for the recombination process, $f_{i}$ depends on the distance between the mesh site (i,j) and the substrate or left electrode, i.e., $x_{i}=a\times i$, and the product vt is the distance that an $O_{\mathrm{ions}}$ travels and $L_{O}$ is the decaying length of the $O_{\mathrm{ions}}$ concentration in the left side of the active layer.

Since the linear model of $T_{J}$ is related to the temperature of CFs [40], $T_{J}$ affects the velocity of $O_{\mathrm{ions}}$ (*v*), defined in (1), and the recombination probability, defined in (3). On the other hand, in this model, the generation probability defined by (2) is not affected by the Joule heating. Therefore, only the temperature $T_{r}$ is considered [23].

$F_{ij}^{nH}$ and $f_{i}$ are defined as [26]:

(4) $$F_{ij}^{nH}=\frac{\left|V_{L}-V_{R}\right|}{L-a\sum_{i=1}^{N}\delta_{Vo,ij}}$$

(5) $$f_{i}=\left\{\begin{array}{cc} 1, & x_{i}\leq vt\\ 0.3, & vt<x_{i}\leq vt+a\\ 0.1, & vt+a<x_{i}\leq vt+3a\\ 0, & x_{i}>vt+3a \end{array}\right.$$

where $V_{L}$ is the voltage applied to the Si-substrate, which is assumed to be equal to the voltage of the bottom aluminum electrode, $V_{R}$ is the voltage of the top aluminum electrode (Figure 1a), $\delta_{Vo,ij}$ is the Kronecker’s delta that can determine if the site (i,j) is a $V_{O}$.

**Figure 3:**
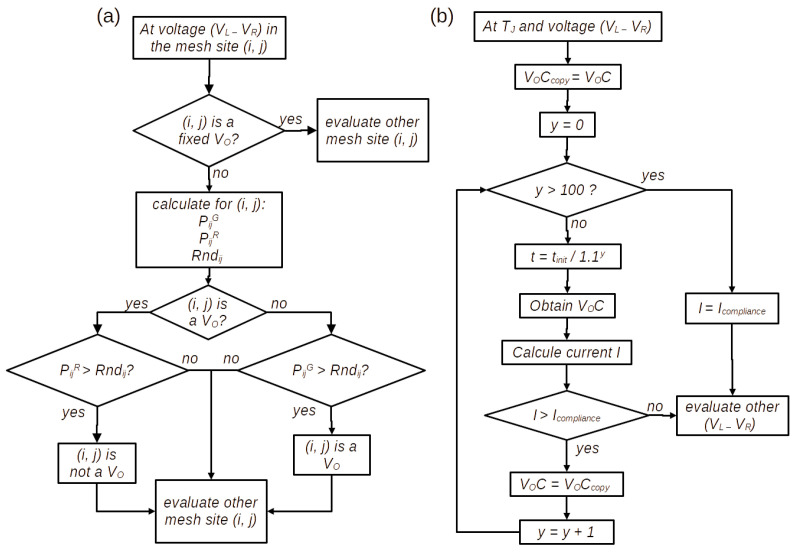
Algorithm of: (**a**) Stochastic process for each (i,j) site. (**b**) Implementation of current compliance.

As we can see in Figure 3a, the stochastic process consists in the following: at each potential difference $\left(V_{L}-V_{R}\right)$ and current *I*, if the site (i,j) is not a fixed $V_{O}$ or Si-NCs site (blue circles in Figure 2), the probability $P_{ij}^{G}$ from (2), $P_{ij}^{R}$ from (3) and a random number ${Rnd}_{ij}$ are calculated. If this site is a $V_{O}$ and the $P_{ij}^{R}$ is greater than ${Rnd}_{ij}$, the site passes to non-$V_{O}$, which indicates it is not a $V_{O}$. If the site is a non-$V_{O}$ and the $P_{ij}^{G}$ is greater than ${Rnd}_{ij}$, the site passes to a $V_{O}$. In some cases, the RSM devices require a current compliance ($I_{compliance}$) to ensure reversible RS and to avoid severe damage in the active material [41]. The control of the current compliance is carried out at a new $\left(V_{L}-V_{R}\right)$ with temperature $T_{J}$ (Joule effect from previous values of current and voltage), as follows (Figure 3b): a copy of $V_{O}$C is saved in $V_{O}C_{\mathrm{copy}}$; then a new $V_{O}$C, due to the initial time ${t=t}_{init}$ used when $I<I_{compliance}$ (Equations (2), (3) and (5)), is obtained by the stochastic process. Then, the current *I* is calculated for this new $V_{O}$C. If the *I* is greater than $I_{compliance}$, the $V_{O}C_{\mathrm{copy}}$ is recovered and $t_{init}$ is reduced by a factor of $1/1.1^{y}$ where *y* increases in one unit. The same algorithm is repeated until $I≅I_{compliance}$ or that $t_{init}$ be reduced to a factor of $1/1.1^{100}$, assuming the $V_{O}$C does not change and that the $I=I_{compliance}$.

## 4. Oxygen Vacancy Configurations to Obtain the Resistive States

The resistive state, in bipolar mode, can be estimated using the Mott hopping model [42] for each different $V_{O}$C obtained at the potential difference $\left(V_{L}-V_{R}\right)$ by calculating the hopping rate of the CF for the row *j* ($R_{j}$) (Figure 2):

(6) $$R_{j}\propto exp\left[\frac{-\left(L-a\sum_{i=1}^{N}\delta_{Vo,ij}\right)}{a_{0}}\right]$$

where $a_{0}$ is the attenuation length of the electron wave function. The largest CF weighs the most since the $V_{O}$ chain will have the smallest distance to the left electrode (Figure 2). Considering the *M* rows, the resistive state $N_{S}$ of each $V_{O}$C is:

(7) $$N_{S}=\frac{Ln\left(\sum_{j=1}^{M}R_{j}\right)}{M}$$

**Figure 4:**
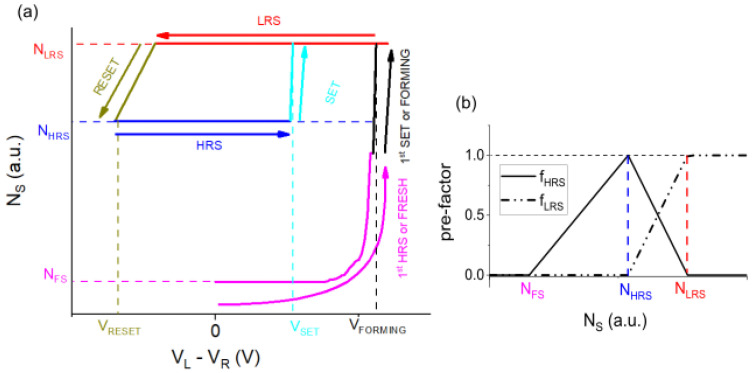
(**a**) The ideal scheme of the resistive state of different $V_{O}$ configurations using the quantity $N_{S}$ through voltage sweeps. (**b**) Definition of pre-factors $f_{HRS}$ and $f_{LRS}$.

The estimation of $N_{S}$ is included in the step “*Calculate current I*” in the flowchart of Figure 3b. An ideal scheme of a closed loop of $N_{S}$ as a function of voltage sweep $\left(V_{L}-V_{R}\right)$ is shown in Figure 4a. We can identify three stable states: FRESH, HRS and LRS and their constant values for each $V_{O}$C, i.e., $N_{FS}$, $N_{HRS}$ and $N_{LRS}$, respectively. Thus, we can see some important values of voltage $\left(V_{L}-V_{R}\right)$: the voltage needed for the FORMING ($V_{FORMING}$), the voltage where the SET ($V_{SET}$) occurs and the voltage that achieve the RESET ($V_{RESET}$).

## 5. Conduction Mechanisms at Different Resistive States

Different works have reported that the conduction mechanism depends on the resistive state, the RS material and the electrodes [43]. Based on the linear pre-factors for different resistive states proposed by Hu et al. [6], we use two factors, $f_{HRS}$ and $f_{LRS}$ that depend on $N_{S}$ and that multiply the current from different conduction mechanisms at HRS and LRS. These factors are defined by the piecewise functions shown in Figure 4b.

For the HRS, the charge transport can be due to the Poole-Frenkel (P-F) mechanism, which accounts for the reduction of potential wells at high electric field $F^{H}$ on the thin oxide layer [44], which is defined as [45]:

(8) $$J_{HRS}=f_{HRS}K_{HRS}q\left|F^{H}\right|\mu N_{C}\exp\left(\frac{\beta \sqrt{\left|F^{H}\right|}-q\phi_{t}}{kT_{r}}\right)$$

where the constant $\beta =\sqrt{q^{3}/\pi \varepsilon }$ with ε as the dielectric permittivity, μ the electron mobility, $N_{C}$ the effective density of states in the conduction band, and $\phi_{t}$ the potential of the trap levels below the conduction band. $K_{HRS}$ is an enhancement factor for the P-F mechanism. For the LRS, the space charge limited current with Frenkel effect (SCLC-F) was used, which takes into account the high $F^{H}$ on the thin oxide layer [46]:

(9) $$J_{LRS}=f_{LRS}K_{LRS}\frac{9}{8}\mu \varepsilon \frac{N_{C}}{N_{S}-N_{FS}}\frac{{\left(F^{H}\right)}^{2}}{L}\exp\left(\frac{0.891\beta \sqrt{\left|F^{H}\right|}-q\phi_{t}}{kT_{J}}\right)$$

where $K_{LRS}$ is an enhancement factor for the SCLC-F. $K_{HRS}$ and $K_{LRS}$ are constant values that must be determined by experimental results because the 2-D model tries to estimate the behavior of the 3-D real devices, and $N_{S}$ is a dynamic value that is affected by the two dimensional $V_{O}$C approximation.

Finally, the current *I* at each voltage $\left(V_{L}-V_{R}\right)$ with the top electrode area *A* is given by:

(10) $$I=\left(J_{HRS}+J_{LRS}\right)A$$

## 6. Simulation and Results

The bipolar RS behavior of Si$O_{2}$/Si-NCs bilayers (BLs) (6 nm thick/6 nm diameter) with an upper layer of Si$O_{2}$ (10 nm thick) was studied using MOS-like devices, as schematized in Figure 1a. Two RSMs were simulated, devices with one and three BLs (labeled as 1-BL and 3-BL, respectively). For our simulation, we use the values given in Table 1.

**Figure 5:**
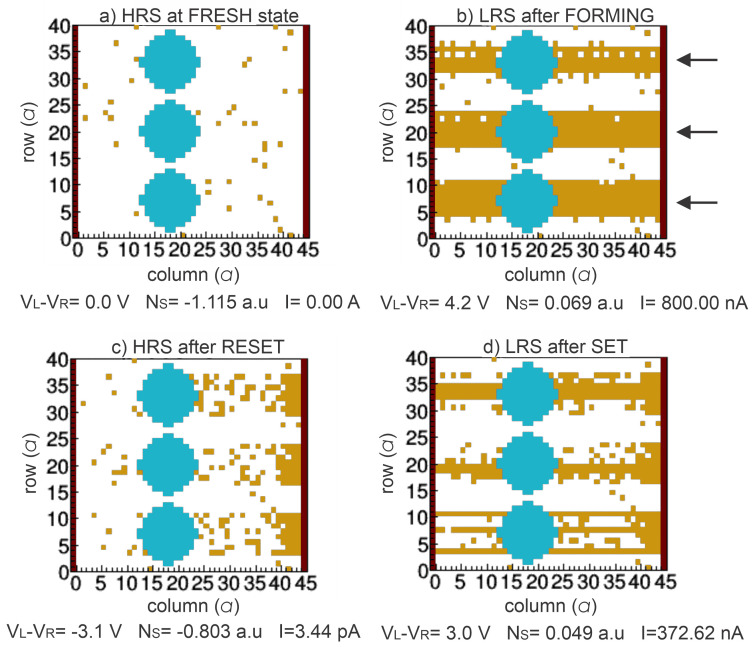
2-D $V_{O}$C for 1 SiO${}_{2}$/Si-NCs bilayer, where blue sites represent Si-NCs and orange sites are oxygen vacancies in the ${\mathrm{SiO}}_{x}$ matrix in the following states: (**a**) First high resistive state (HRS) or fresh without any electrical stress and some $V_{O}$ randomly distributed in a 2-D lattice. (**b**) Low resistive state (LRS) after first soft breakdown, i.e., FORMING process. (**c**) HRS after RESET process. (**d**) LRS after SET process.

**Figure 6:**
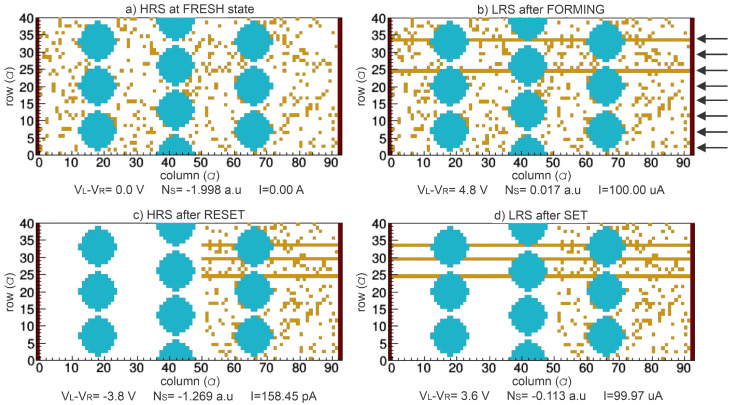
2-D $V_{O}$C for 3 SiO${}_{2}$/Si-NCs bilayers, where blue sites represent Si-NCs and orange sites are oxygen vacancies in ${\mathrm{SiO}}_{x}$ matrix in the following states: (**a**) First high resistive state (HRS) or fresh without any electrical stress and some $V_{O}$ randomly distributed in a 2-D lattice. (**b**) Low resistive state (LRS) after first soft breakdown, i.e., FORMING process. (**c**) HRS after RESET process. (**d**) LRS after SET process.

Figure 5 and Figure 6 show the different $V_{O}$Cs obtained for the different resistive states in 1-BL and 3-BL devices, respectively. For FRESH devices (Figure 5a and Figure 6a), they behave with an HRS state with some initial $V_{O}$ ($V_{\mathrm{Oinit}}$). Once the device is electrically biased, CFs are preferentially formed through Si-NCs or fixed $V_{O}$ to obtain the first LRS (SET or FORMING), as shown in Figure 5b and Figure 6b. The $V_{O}$s are recombined with $O_{\mathrm{ions}}$ near the left electrode when an opposite bias is applied to the devices inducing the HRS (RESET), see Figure 5c and Figure 6c. Finally, other CFs can be formed because additional $V_{O}$s can be generated and recombined during a voltage sweep cycle, Figure 5d and Figure 6d.

The high $F_{ij}^{nH}$ defined in (4) promotes the formation of CFs near the Si-NCs because they are assumed to be fixed groups of $V_{O}$ increasing $P_{ij}^{G}$ (defined in (2)). In other words, the presence of fixed $V_{O}$, which acts as a conductive material, reduces the equivalent distance between electrodes inducing a high electric field. As observed in Figure 5b and Figure 6b, a larger quantity of CFs are formed in the 1-BL device than the 3-BL device since a higher electric field is obtained for the thinner oxide of 1-BL device (Table 1). In these devices, the CFs at LRS can be preferentially formed in the rows where a greater number of fixed $V_{O}$s exist; that is, where the Si-NCs are present as indicated by the arrows in Figure 5b and Figure 6b. In fact, experimental evidence of this effect has been reported before [12].

**Figure 7:**
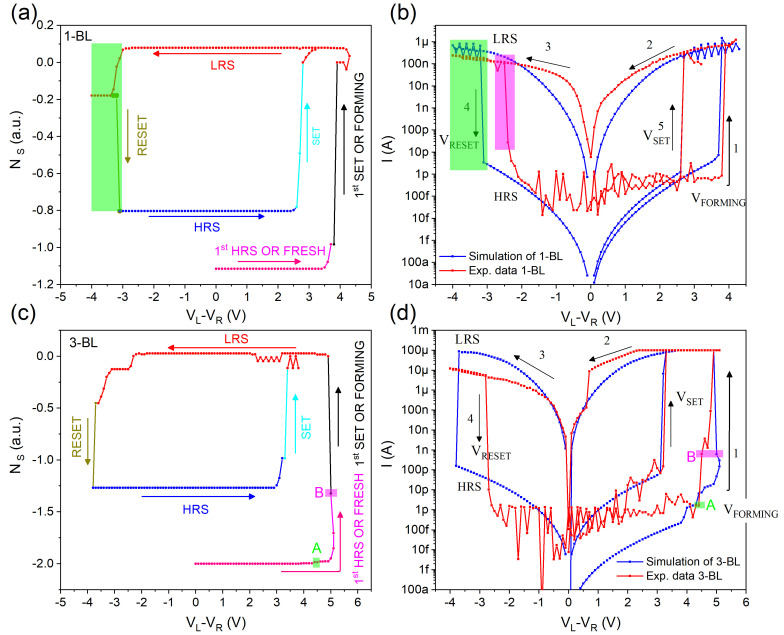
Simulation and experimental data of 1-BL and 3-BL devices: (**a**) Resistive state $N_{S}$ and (**b**) I-V of 1-BL device. (**c**) Resistive state $N_{S}$ and (**d**) I-V of 3-BL device.

The simulated $N_{S}$, as well as the experimental and simulated I-V curves obtained for 1-BL- and 3-BL-based memristors are shown in Figure 7. A detailed explanation of FORMING, RESET and SET processes are presented below.

### 6.1. FORMING and SET Process

The $V_{FORMING}$ is modulated by the initial number of $V_{O}$s ($V_{O}$init) randomly placed within the active material. The 3-BL device is formed by two additional Si$O_{2}$/Si-NCs bilayers compared to the 1-BL device. Therefore, a higher concentration of defects and $V_{O}$ need to be considered for this device [47]. The $V_{\mathrm{Oinit}}$ value was 40 for 1-BL device whereas $V_{\mathrm{Oinit}}$ = 400 was considered for the 3-BL to adjust the experimental $V_{FORMING}$ (Table 1).

**Figure 8:**
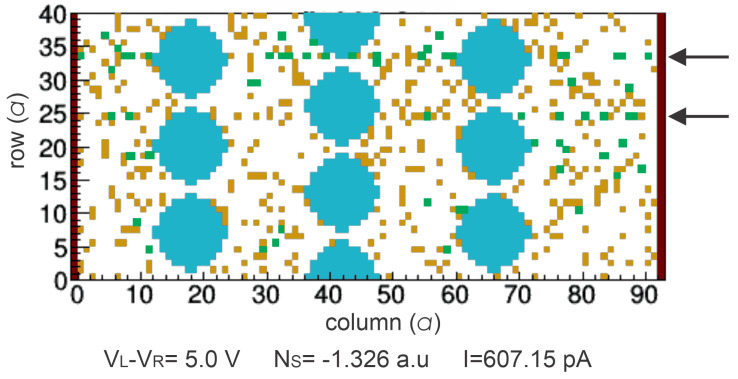
Simulation of the $V_{O}$C for 3-BL device near the experimental IRS step (627.07 pA at 4.5 V (point B in Figure 7c,d)). The green sites are the new $V_{O}$s respect to $V_{O}$C simulated at 4.3 V (point A in Figure 7c,d). Arrows indicate the $V_{O}$s that will form complete CFs in rows j=25 and j=34 at LRS (Figure 6b).

As we can see in the Figure 7b,d, the simulation of the FORMING process in both devices adjust to their experimental I-V data. The experimental FORMING process in the 1-BL device is observed as an abrupt change of current at $V_{FORMING}=3.9$ V while the simulated one occurs at 3.8 V (Figure 7a,b). For the 3-BL device, the FORMING process exhibits an intermediate resistive state (IRS) level, Figure 7d, until the current increases to the highest current value at $V_{FORMING}=4.9$ V (Figure 7c,d). As we can see, the simulated data fit well to the experimental data at the same $V_{FORMING}$. The experimental IRS is obtained when the current jumps from 1.61 pA at 4.4 V to 627.07 pA at 4.5 V. Figure 8 shows the simulation of the $V_{O}$C in this current jump. As we can see, new $V_{O}$s (green dots) are generated as the simulated voltage increases from 4.3 V (indicated by A in Figure 7d) to 5.0 V (indicated by B in Figure 7d). Then, the IRS level can be explained as a result of new $V_{O}$, preferentially generated at rows j=25 and 34, as indicated by the arrows, which will form the complete CF to obtain the LRS (Figure 6b).

The simulation of the 3-BL device (Figure 7c,d) also shows the change from the HRS state to LRS (FORMING). This process occurs when the voltage sweep returns to 0V (sweep 2) at $V_{FORMING}=4.9$ V, while the experimental FORMING is obtained for the voltage sweep from 0 V to 5.1 V (sweep 1) at $V_{FORMING}=4.9$ V (Figure 7d). This difference between the experimental and simulated FORMING process can be explained by the stochastic nature of the RS phenomena [48].

The simulation of the SET process for the 1-BL and 3-BL devices fits well to the experimental I-V data, as observed in Figure 7b,d, respectively. For the 1-BL device, the experimental and simulated $V_{SET}$ is about 2.7 V while for the 3-BL device, the simulated $V_{SET}$ is about 3.2 V, which is near the experimental $V_{SET}$ at 3.3 V.

### 6.2. RESET Process

Once the devices are in the ON state (LRS), an opposite voltage sweep produces the RESET (OFF) process. However, the simulations of this process for both 1-BL and 3-BL devices do not fit to the experimental RESET. Experimental $V_{RESET}$ values were −2.4 V and −2.7 V for the 1-BL and 3-BL devices, respectively. Meanwhile, simulated $V_{RESET}$ values were −3.1 V and −3.8 V for the 1-BL and 3-BL devices, respectively. This difference can be related to the stochastic process of the generation and recombination of $V_{O}$ during the RESET event. In fact, the RESET process has been reported to be dependent on thermal noise and nonlinear relaxation phenomena [49], which is not included in the present model.

**Figure 9:**
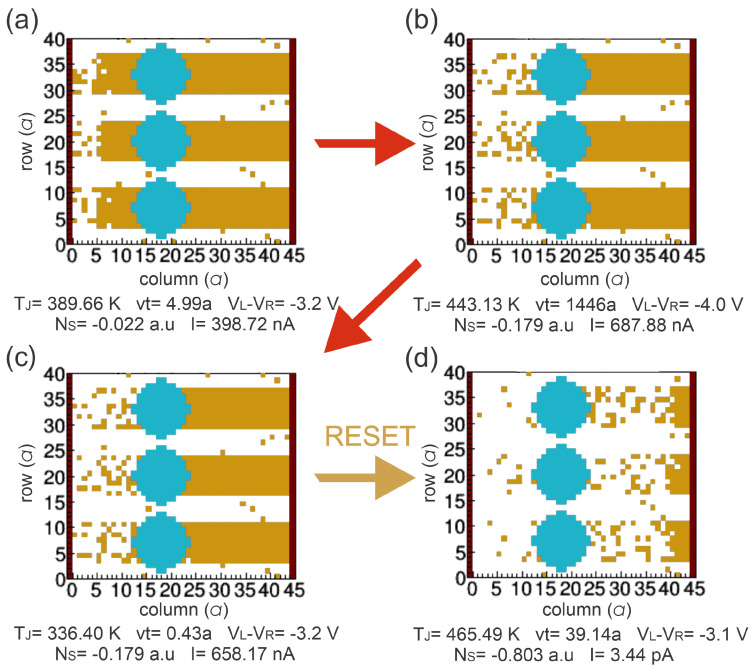
Simulation of RESET process during the negative voltage sweep of 1-BL device: (**a**) At −3.2 V during the sweep 3 of Figure 7b. (**b**) At −4.0 V. (**c**) At −3.2 V during the sweep 4 of Figure 7b. (**d**) At $V_{RESET}=-3.1$ V during the sweep 4 of Figure 7b.

As we can see in Figure 7b, the experimental RESET process of the 1-BL device was obtained when the voltage is swept from −4.0 V to 0 V (pink zone). The simulation of this process also shows the RESET at the same voltage sweep (green zone). Figure 9 shows the evolution of the $V_{O}$Cs simulated for a voltage cycle from −3.2 V to −4.0 V (sweep 3) to −3.1 V (sweep 4) to explain the RESET process for the 1-BL device. When the voltage increases from −3.2 V to −4.0 V (Figure 9a,b), the $V_{O}$s near to the substrate (left electrode) recombine reducing the $N_{S}$ from −0.022 a.u. at $\left(V_{L}-V_{R}\right)=-3.2$ V to −0.179 a.u. at −4.0 V, but keep the device at the LRS. As we know, the Joule heating is an important parameter in the deoxidation–oxidation model. Then, a change in the temperature produced by the Joule heating affects the distance (vt) that $O_{\mathrm{ions}}$ can travel. If *v* is high enough, the $O_{\mathrm{ions}}$ do not recombine with $V_{O}$s. The *v* of the $O_{\mathrm{ions}}$ at −4.0 V, as calculated by (1) increases due to a $T_{J}=443.13$ K. Therefore, the distance that $O_{\mathrm{ions}}$ can travel is about vt≅1446a, which is higher than the thickness of the active layer L=45a (Figure 9b). The $V_{O}$C remains unchanged as the voltage decreases from −4.0 V to −3.2 V (Figure 9b,c). However, the $T_{J}$ value decreases from 443.13 K to 336.4 K resulting in the decreasing of the distance that $O_{\mathrm{ions}}$ travel to vt=0.43a. Consequently, the $O_{\mathrm{ions}}$ do not recombine with those $V_{O}$s far away from the left electrode. As the voltage reduces from −3.2 V to −3.1 V (Figure 9c,d), an abrupt current drop is obtained from 658.17 nA to 3.44 pA (RESET). This strong current drop is related to an increased temperature $T_{J}$ from 336.4 K to 465.49 K, which results in a vt=39a that allows the $V_{O}$s to recombine, producing the RS from LRS a HRS (Figure 9d).

**Figure 10:**
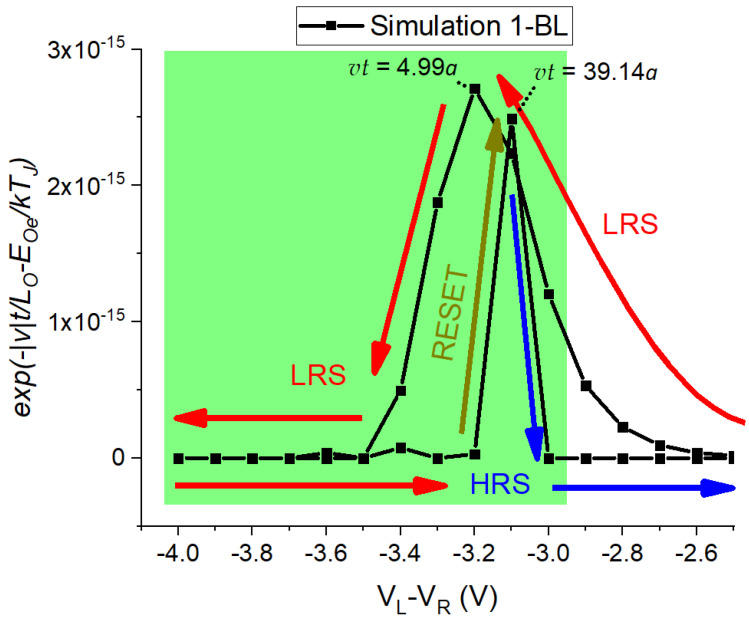
Exponential factor for the calculation of $P_{R}$ used in (3) for the simulation of the RESET of 1-BL device.

As observed, the simulation of the RESET process depends on *v* of the $O_{\mathrm{ions}}$ and the Joule heating since both control the recombination probability, as defined by (3). In fact, Figure 10 shows the exponential factor of the recombination probability for the 1-BL device. A maximum value is observed at $\left(V_{L}-V_{R}\right)=-3.2$ V as the voltage sweep increases from −2.5 V to −4.0 V. Nevertheless, the distance vt≅5a that $O_{\mathrm{ions}}$ travel is not enough to obtain the HRS (Figure 9a). A second maximum value of the exponential factor is obtained at about −3.1 V when the voltage sweep reduces from −4.0 V to −2.5 V. This second maximum produces the RESET process due to the distance vt≅39a, which is similar to the thickness of the active material L=45a. For $\left(V_{L}-V_{R}\right)\leq -3.0$ V, the device remains at the HRS, decreasing the $T_{J}$ and the *v* values.

## 7. Conclusions

In this work, the 2-D simulation of the RS processes of Si-NCs/SiO${}_{2}$ ML has been analyzed. The Si-NCs were assumed as agglomerates of fixed $V_{O}$ which are not affected for deoxidation–oxidation process. The semiempirical model of VCM is simplified using the mathematical expression based on the Mott hopping model to calculate the resistive state considering the $V_{O}$C. This approximation allows for the use of a different conduction mechanism. It was found that P-F and SCLC-F fit to the experimental HRS and LRS for both 1-BL and 3-BL devices. The simulation of Si$O_{2}$/Si-NCs ML demonstrates that the CFs are preferentially formed near the Si-NCs enhancing the LRS (ON). The simulated voltage fits well to experimental I-V data to achieve the FORMING and SET. An IRS is observed in the FORMING process of the 3-BL device, which is explained by the preferential generation of $V_{O}$ in the sites that form the complete CFs, through Si-NCs. The deoxidation–oxidation model shows that the RESET process depends on the oxygen ion velocity since it is affected by Joule heating.

## Figures and Tables

**Table 1:** Values used for simulations.

Parameter	Symbol	1-BL	3-BL
Time to generate or recombine $V_{O}$	$t_{init}$	5 μs	
Vibration frequency of the $O_{\mathrm{ion}}$	$1/t_{0}$	$10^{13}$ Hz	
Migration barrier from oxygen equilibrium	$E_{Oe}$	1 eV	
Enhancement coefficient of generation probability during SET and FORMING	γ	4.6	
Enhancement coefficient of generation probability during RESET	γ	0.4	
Thickness of oxide	*L*	22 nm	46 nm
Initial number of $V_{O}$	$V_{\mathrm{Oinit}}$	40	400
Mesh size	*a*	0.5 nm	
Coefficient for the recombination	$\beta_{R}$	$6\times 10^{6}$	
Decaying length of the $O_{\mathrm{ion}}$ concentration	$L_{O}$	6.3×a	
Height of the potential barriers during the migration of $O_{\mathrm{ion}}$	$E_{Oe}$	1 eV	
Enhancement coefficient for the drift of $O_{\mathrm{ion}}$	$\varphi_{drift}$	8	
Attenuation length of the electron wave function	$a_{0}$	0.33 nm	
Enhancement factor for P-F mechanism	$K_{HRS}$	$8\times 10^{-23}$ a.u.	$8\times 10^{-20}$ a.u.
Electron mobility	μ	$1.45\times 10^{3}$ cm${}^{2}$/Vs	
Effective density of states in the conduction band	$N_{C}$	$2.86\times 10^{19}$ /cm${}^{3}$	
Electrical permittivity	ε	$11.9\left(8.85\times 10^{-14}\right)$ F/cm	
Potential of the trap levels for charge conductivity	$\phi_{t}$	0.1 V	
Enhancement factor of SCLC-F	$K_{LRS}$	$3\times 10^{-35}$ a.u.	$1\times 10^{-30}$ a.u.
Device area	*A*	1 mm${}^{2}$	
Thermal resistance of CFs	$R_{th}$	$8\times 10^{7}$ K/W	$9\times 10^{5}$ K/W
$N_{S}$ at LRS	$N_{LRS}$	0.2	
$N_{S}$ at HRS	$N_{HRS}$	−0.7	−1
$N_{S}$ at fresh state	$N_{FS}$	−1.4	−2

## Data Availability

Not applicable.
